# Requirement of ClpX for CtsR dissociation from its operator elements upon heat stress in *Bacillus subtilis*

**DOI:** 10.3389/fmicb.2025.1699655

**Published:** 2025-11-07

**Authors:** Marco Harms, Chelsea Kaden, Larissa M. Busch, Vishnu M. Dhople, Ulf Gerth, Manuela Gesell Salazar, Stephan Michalik, Zhanetta Zhatarova, Uwe Völker, Alexander Reder

**Affiliations:** 1 Department of Functional Genomics, Interfaculty Institute for Genetics and Functional Genomics, University Medicine Greifswald, Greifswald, Germany; 2 Institute of Microbiology, University of Greifswald, Greifswald, Germany; 3 Department of Medicine, Biochemical/Pharmacological Centre, Philipps-University Marburg, Marburg, Germany

**Keywords:** *Bacillus subtilis*, class III heat-shock regulon, ClpX, CtsR, *clpE*

## Abstract

A sudden increase in temperature triggers *Bacillus subtilis* to activate expression of stress-specific heat shock proteins of the CtsR (class three stress gene repressor) regulon to withstand the adverse conditions. Key members of this regulon, such as ATPases, proteolytic subunits and their adaptors, which can assemble to the functional Clp protease system, perform crucial roles in maintaining cellular proteostasis, while their transcription is repressed by CtsR during vegetative growth. Upon heat shock, a conformational change in a thermosensing glycine-rich loop causes CtsR to detach from its DNA operators, enabling the transcriptional activation of the regulon. Novel data from a *clpX*-deficient strain demonstrated that in addition, the presence of the ATPase ClpX is essential for the CtsR dissociation from its DNA binding site. To further elucidate this role of ClpX, we constructed a conditional *clpX* strain, in which *clpX* induction is decoupled from its native transcriptional control. This conditional expression system mimicked a *clpX-*deficient phenotype under non-inducing conditions and restored the wild-type phenotype upon induction. Our results indicate that the full induction of the CtsR regulon, particularly *clpE*, requires both heat and the presence of ClpX, thereby extending the current model for the transcriptional activation of genes repressed by CtsR.

## 1 Introduction

Heat poses a significant threat to the cellular integrity of the Gram-positive bacterium *Bacillus subtilis*, especially in its natural soil habitat, where temperature fluctuations are frequent. Elevated temperatures can disrupt cellular processes by protein misfolding and aggregation, which can severely affect survival. To counteract these adverse effects, *B. subtilis* has evolved an intricate regulatory heat shock stimulon that orchestrates the adaptive heat response [reviewed in Schumann (2003)]. Key regulators of this system include the alternative sigma factor SigB (Price et al., 2001) and repressors such as HrcA (Schulz and Schumann, 1996; Yuan and Wong, 1995) and CtsR (Derré et al., 1999b; Krüger and Hecker, 1998) as well as the response regulator CssR (Darmon et al., 2002; Hyyryläinen et al., 2001).

The degradation of misfolded or damaged proteins is crucial for protein homeostasis especially under heat stress conditions. This process is mediated by the gene products of the CtsR regulon, namely key members of the AAA + superfamily (ATPases associated with various cellular activities). Chaperones and proteases within this family play pivotal roles in maintaining protein quality, while their function relies on ATP hydrolysis (Erzberger and Berger, 2006; Gottesman et al., 1997a; Pak et al., 1999). These bacterial AAA + complexes typically comprise an unfoldase and a protease subunit, forming hexameric and heptameric rings respectively, that assemble into barrel-like protease complexes (Martin et al., 2005). The Clp (caseinolytic proteases) proteins, integral members of the AAA + family, function either as chaperones assisting protein refolding (Gottesman et al., 1997b; Schirmer et al., 1996; Wickner et al., 1999) or in interaction with the proteolytic subunit as protease systems, mediating degradation (Hinnerwisch et al., 2005; Weber-Ban et al., 1999). In *B. subtilis*, the ATP-dependent Clp protease proteolytic subunit (ClpP) (Kock et al., 2004) associates with one of the ATPase subunits ClpC, ClpX or ClpE (Gerth et al., 2004). The specific ATPase subunit dictates substrate specificity, determining which proteins are targeted for degradation (Hoskins et al., 1998; Levchenko et al., 1997).

**FIGURE 1:**
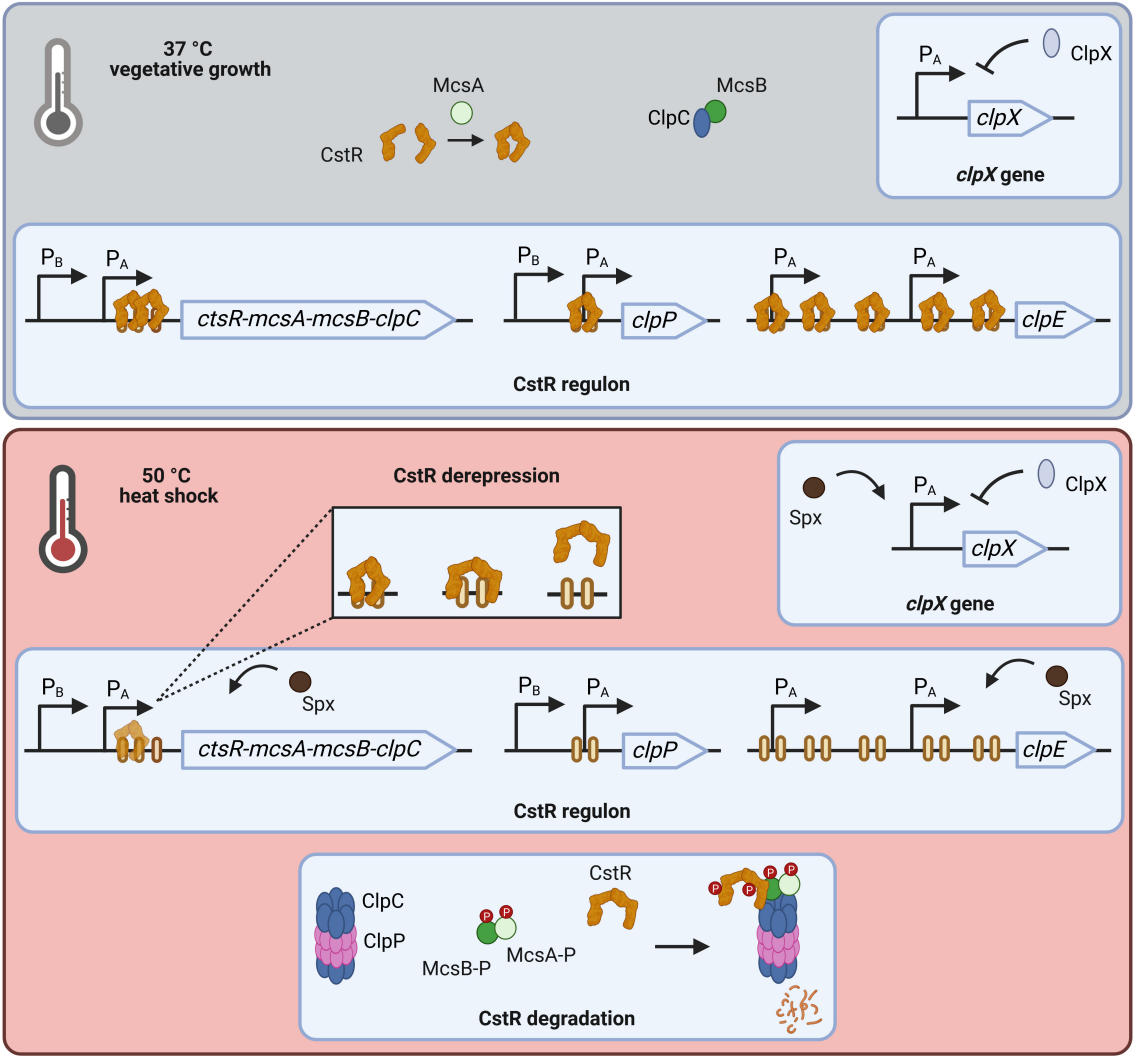
Regulation of *clp* gene expression in *Bacillus subtilis*. During vegetative growth CtsR represses transcription of its regulon. McsA putatively assists CtsR dimerization while activity of McsB is abolished by ClpC binding. Transcription of *clpX* as major ATPase is negatively autoregulated. These growth conditions result in expression of low basal level of *clpC* operon members and *clpP*, as well as *clpX*. In contrast, *clpE* is tightly repressed by CtsR. Upon heat stress CtsR changes conformation, which leads to its dissociation from DNA binding sites. Assembly of ClpCP releases McsB. McsA activates McsB leading to phosphorylation of both proteins and subsequent phosphorylation of free, partly unfolded CtsR. The phosphorylated CtsR-McsB-McsA complex is targeted for degradation by ClpCP. As consequence, transcription of now derepressed regulon members is initiated, causing a strong and transient expression. This induction is further accelerated by action of the activator Spx. [Created in BioRender. Völker, U. (2025) https://BioRender.com/h9h78fa].

During vegetative growth, the dimeric helix-turn-helix (HTH) CtsR repressor protein prevents the transcription of its regulon members by binding to a highly conserved heptanucleotide direct repeat (A/GGTCAAA) within the −35 and −10 core promoter regions (Derré et al., 1999a,b, 2000). Cooperative binding of CtsR inhibits transcriptional initiation by the RNA polymerase (Derré et al., 2000). The CtsR regulated genes include the *clpC* operon (*ctsR*-*mcsA*-*mcsB*-*clpC*), as well as the *clpP* and *clpE* gene. Each of these elements is controlled by multiple promoters: the *clpC* operon and *clpP* are preceded by one σ*^B^*-type and σ*^A^*-type promoter, while the *clpE* gene is driven by two σ*^A^*-type promoters. Notably, the number of CtsR binding sites varies between the regulated genes, indicating differences in repression tightness by CtsR (see Figure 1; Derré et al., 1999a,b; Gerth et al., 1998; Krüger et al., 1996, 2000). At 37 °C, the *clpC operon* and *clpP* genes are expressed only at low basal levels due to CtsR repression and the *clpE* gene even remains completely repressed by five CtsR binding sites. CtsR activity is modulated in a feedback-loop by the gene products of its regulon. McsA, encoded by the second gene of the *clpC operon*, promotes CtsR binding to the operator and repression of target genes by supporting CtsR dimerization (Krüger et al., 2001). Moreover, ClpC is thought to enhance CtsR activity, putatively by assisting CtsR folding or dimerization (Derré et al., 1999b,^2000^). In contrast, McsB is kept inactive by interaction with ClpC (Kirstein et al., 2005).

Sudden temperature upshifts lead to transcriptional activation of the CtsR regulon. In particular, loss of CtsR DNA-binding affinity is triggered by a heat-dependent conformational change of CtsR within the glycine-rich loop adjacent to the helix-turn-helix (HTH) domain (Derré et al., 2000; Elsholz et al., 2010). Subsequent, degradation of unbound CtsR is regulated by a sophisticated two-step mechanism. Formation of the ClpCP protease complex releases the arginine kinase McsB, which is kept inactive by interaction with ClpC at non-heat-stress temperatures (Elsholz et al., 2011; Kirstein et al., 2005, 2007; Lu et al., 2025). Heat-activated McsA stimulates the protein kinase activity of McsB which results in the phosphorylation of the McsA-McsB complex (Kirstein et al., 2005). This complex phosphorylates primarily free CtsR, which is due to the conformational change partly unfolded, on conserved arginine residues (Fuhrmann et al., 2009; Trentini et al., 2016). Phosphorylation labels CtsR for ClpCP-dependent degradation, and simultaneously, phosphorylation in general promotes assembly of the ClpCP complex (Krüger et al., 2001; Miethke et al., 2006; Trentini et al., 2016). Additional degradation of CtsR by ClpEP is described (Miethke et al., 2006). Induction of CtsR regulated genes is transient, leading to elevated transcript and protein level with *clpE* being the most affected due to the number of CtsR binding sites (Derré et al., 1999a; Gerth et al., 2004). Further positive regulation is achieved by the action of Spx on *clpC* operon and the *clpE* gene (Rochat et al., 2012). However, *clpX* transcription is negatively autoregulated and can be activated by the transcriptional activator Spx under stress conditions (Gerth et al., 2004; Rochat et al., 2012). ClpX functions as the major ATPase at 37°C and is therefore responsible for degradation of CtsR *via* the ClpXP proteolytic complex (Derré et al., 2000). Although transcription of *clpX* is upregulated upon heat stress, protein levels remain largely unchanged (Gerth et al., 1996, 2004).

Of note, the regulation of the CtsR-independent monocistronic *clpX* gene differs from the other *clp* genes. Like the CtsR-regulated genes, *clpX* is preceded by a σ*^A^*-type promoter (Gerth et al., 1996, 2004).

In this study, we report that both the presence of ClpX and heat are prerequisites for the CtsR dissociation of its binding site, thus achieving full induction of the CtsR regulon. Given the experimental data of the newly generated in-frame *clpX* deletional and conditional *clpX* strain, we hypothesize that the chaperone function of ClpX supports the conformational change required for CtsR to detach from its heptanucleotide operator during heat stress.

## 2 Material and methods

### 2.1 Generation of mutant strains

Genetic modifications were incorporated into the chromosome of *B. subtilis* BSB1 (Nicolas et al., 2012). To generate the mutant strains (BZZ01-BZZ05, see Table 1), a modified PCR protocol (Wach, 1996) was utilized. This method resulted in generation of linear DNA fragments including the resistance cassette, replacing the target gene and homologous up- and downstream fragments of the desired mutation site. Obtained PCR products were purified and fused according to a procedure described in Dittmar et al. (2020). Used primers are detailed in Supplementary Table 1: Primer pairs containing either “up” or “do” are considered primer pairs for the flanking homologous fragments, primer pairs tagged with “Res” were used to amplify the resistance cassette. For the transformation of *B. subtilis* BSB1, modified competence media (MC) according to Smith et al. (2024) was used. A total of 20 ml MC medium was inoculated to OD_500_ of 0.05 with an exponentially growing overnight LB culture and cultivated at 37°C, 220 rpm (Innova 4230 Refrigerated Incubator Shaker, New Brunswick). When the MC culture reached an OD_500_ of 2.0, 2 ml were transferred to another cultivation tube containing 500 μg of respective PCR fragment and further incubated at 90 rpm. After 1 h, 1 ml LB medium was added and cultivation continued for 3 h. The respective mutants were selected on LB agar plates containing either 1 μg/ml erythromycin, 5 μg/ml kanamycin, 5 μg/ml phleomycin, 5 μg/ml chloramphenicol or 200 μg/ml spectinomycin. Chromosomal DNA was isolated and mutant clones were verified by Sanger sequencing. The strains BZZ01, BZZ03, BZZ04 and BZZ05 were designed as in-frame deletions, in which the resistance cassette replaces the target at its original chromosomal locus. In case of strain BZZ02, the target gene *clpE* together with its preceding promoter region was replaced by a phleomycin cassette with an artificial optimal SigA promoter (TTGACA-17bp-TATAAT) and Shine-Dalgarno sequence (GAAGGAGG). For the conditional *clpX* mutant (BCSH01), the native SigA promoter of the *clpX* gene was replaced to an artificial SigA promoter (TTGACT-17bp-TATAAT) that closely mimics the SigA consensus promoter architecture (Helmann, 1995) and is embedded with three tetracycline operators (P_*TRE*_). Furthermore, *tetR* gene was cloned upstream of the chloramphenicol resistance cassette. Expression of the repressor TetR results in binding to its operators upstream of *clpX* and consequent repression of transcriptional initiation under vegetative growth conditions. Addition of anhydrotetracycline, which sequesters TetR by binding, allowed *clpX* transcription and therefore selective and independent control of the ClpX protein level, regardless of its natural regulation. Detailed information on the construction can be found in Figure 4A. BCSH04 (conditional *clpX*Δ*ctsR*) was generated by amplifying a linear DNA fragment of chromosomal DNA of BCSH01 with the primers 1_cond_ClpX_up_for and 6_cond_ClpX_do_rev followed by transformation in the *B. subtilis* strain BZZ05.

**TABLE 1:** *Bacillus subtilis* strains used in the experiments.

Strain	Relevant features	References	Comment
*B. subtilis BSB1*	*trp* ^+^	Nicolas et al., 2012	–
*B*ZZ01	*trp^+^, clpC:km^R^*	This study	Primers in Supplementary Table 1
*B*ZZ02	*trp^+^, clpE:phleo^R^*	This study	Primers in Supplementary Table 1
*B*ZZ03	*trp^+^, clpP:spec^R^*	This study	Primers in Supplementary Table 1
*B*ZZ04	*trp^+^, clpX:erm^R^*	This study	Primers in Supplementary Table 1
*B*ZZ05	*trp^+^, ctsR:km^R^*	This study	Primers in Supplementary Table 1
*BCSH01*	*trp^+^, tig-tetR-cat^R^-P_TRE_-clpX*	This study	Primers in Supplementary Table 1
*BCSH04*	*trp^+^, ctsR:km^R^, trp^+^, tig-tetR-cat^R^-P_TRE_-clpX*	This study	Primers in Supplementary Table 1

### 2.2 Cultivation for stress experiments and cell sampling

Cultivations were conducted in at least three biological replicates, in a darkened air incubator at 220 rpm and 37 °C (Innova 4230 Refrigerated Incubator Shaker, New Brunswick). A serial dilution was generated by adding 500 μl of the glycerol stock in 5 ml of Lysogeny Broth (LB) medium in disposable reagent tubes (13 ml) and mixed thoroughly. Next, 700 μl was transferred into the second disposable reagent tube, and the process was repeated up to the 12*^th^* dilution and incubated for a maximum of 9 h. On the following day, precultures were inoculated with exponentially growing overnight cultures (OD_540*nm*_ = 0.6–1.0) in 30 ml of pre-warmed LB medium to a starting OD_540*nm*_ of 0.05 and incubated further. As soon as the preculture reached exponential growth, the appropriate volume was transferred to a pre-warmed reaction tube (15 ml), centrifuged at 2,400 × *g* at room temperature (RT) for 1 min. The pellet was resuspended in 100 ml Spizizen’s minimal medium (Harwood and Cutting, 1990) with 0.01% yeast extract to a starting OD_500*nm*_ of 0.05 to inoculate the main culture. In the case for the respective condition of BCSH01, 30 min prior to reaching the OD_500*nm*_ of 0.4 in the main culture, 20 ng/ml anhydrotetracycline (aTc) was added to induce ClpX protein concentrations comparable to those of the wild-type. Once the cultures reached an optical density of OD_500 *nm*_ = 0.4, 16 OD units were harvested, with 8 OD allocated for RNA and the other 8 OD for protein preparation. Harvest of a defined number of OD units enables sampling of similar cell, RNA and protein content for further processing. Samples were immediately cooled down with liquid nitrogen, centrifuged at 10.000 × *g* and 4 °C for 3 min. Supernatant was discarded and cell pellets were stored at −70 °C. Main culture flasks were immediately transferred into a 50 °C water bath (OLS Aqua Pro, GRANT) and incubated at 150 rpm linear shaking. After 10 and 30 min, additional samples were collected as described above.

### 2.3 RNA isolation

RNA-Isolation from the harvested cell pellets was achieved according to Harms et al. (2025) In brief, cells were mechanically disrupted using the Dismembrator (Retsch) bead mill and RNA was subsequently extracted using the acid phenol method described by Majumdar et al. (1991).

### 2.4 Quantitative near-infrared (NIR) Northern blot analysis

Near-infrared-Northern blots were performed according to the protocol supplied by ProTec Diagnostics GmbH. Transcript sizes were determined using the RNA TRUE Ladder (ProTec Diagnostics). To assess RNA quality and validate equal loading, the blots were stained with methylene blue. A *clpE* RNA probe was biotin-labeled by *in vitro* transcription using the MEGAscript™ T7 Transcription Kit (Invitrogen) and a gene-specific PCR product with a T7 promoter (for primer see Supplementary Table 1). For fluorescence detection of the Northern blot signal the Odyssey^®^ CLx imaging system (LICORbio Biosciences) was used. Northern blots were performed as three biological replicates. For better comparison, ratios of detected signals were calculated by setting the wildtype signal after heat stress (t_10_) to 100% as reference induction.

### 2.5 Quantitative near-infrared (NIR) Western blot analysis

By using an SDS-PAGE (Mini-Protean), 4 μg of the protein lysate were loaded into each lane and separated. Transfer of the separated proteins to a polyvinylidene difluoride (PVDF) membrane was achieved by the protocol of Gerth et al. (2004). Detection of NIR Western blots was performed following the protocol of LICORbio Biosciences (Near-Infrared Western Blot Detection Protocol, 2025), using the Odyssey^®^ CLx imaging system (LICORbio Biosciences). A polyclonal antibody against ClpX (dilution 1:5.000) as described in (Gerth et al., 2004) was used.

### 2.6 Proteome analysis

Protein lysates were generated as described previously (Harms et al., 2024). Briefly, cell pellets were disrupted using the Dismembrator bead mill (Retsch) followed by nuclease treatment, ultra-sonication and centrifugation. Protein lysates were precipitated according to the protocol described by Nickerson and Doucette (2020) and resolubilized in fresh 20 mM HEPES buffer (pH 8.0) with 1% SDS. Precipitation was performed for buffer exchange, since components of the cultivation media would interfere with the protein concentration determination using the Micro BCA Protein Assay Kit (Thermo Fisher Scientific) according to Reder et al. (2024).

For the global label free proteome analyses, the samples of the in-frame mutants were prepared using the SP3 purification and digestion protocol as previously described (Ganske et al., 2024) and the samples of the conditional mutant approach were prepared using the automated SP3 purification and digestion as established in Reder et al. (2024) using a modified OT-2 liquid handling robot (Opentrons).

Data independent acquisition (DIA) mass spectrometry (MS) measurements were conducted separately for the four biological replicates of the in-frame mutant data and for the three biological replicates of the conditional mutant data.

For the in-frame mutant data, LC-MS/MS analyses were performed using an Orbitrap Exploris 480 mass spectrometer in combination with an UltiMate 3000 RSLC nano system (both Thermo Fisher Scientific). Detailed specifications of the LC-MS/MS parameters can be found in the Supplementary Table 2. For the conditional mutant data, measurement was carried out with a Bruker TIMS TOF HT (Bruker Daltonics GmbH) coupled with Evosep One liquid chromatography system using Evosep Pure tips (Evosep Biosystems). For specifications see Supplementary Table 3.

Data analysis of the DIA experiments were conducted using Spectronaut version 16 (Biognosys AG) and local normalization for the in-frame mutant data. Detailed search parameters can be found in Supplementary Table 4. For the conditional mutant data set, data analysis was performed using Spectronaut version 20 and median-median normalized. Detailed search parameters are provided in Supplementary Table 5.

Subsequently, the obtained Spectronaut-data was processed using the SpectroPipeR pipeline (Michalik et al., 2025) for quality assessment and MaxLFQ protein level estimation (Cox et al., 2014). Outlier samples were removed manually from the data sets (Supplementary Table 6), based on evaluation of principle component analyses. Proteins were considered identified if detected with at least two peptides. Global statistical testing was performed on peptide level using the reproducibility-optimized peptide change averaging (ROPECA) test (Suomi and Elo, 2017). The results are provided in Supplementary Table 7 and serve as supporting information.

For visualization purposes, protein level estimations of mass spectrometry measurements were normalized to the mean of heat stressed wild-type t_10_ sample protein level estimation. Plots were generated in R v2024.12.1 + 563 using the tidyverse framework v2.0.0 (Wickham et al., 2019).

The mass spectrometry proteomics data for both measurements (in-frame mutants and conditional mutants) as well as the protein databases have been deposited to the ProteomeXchange Consortium *via* the MassIVE partner repository with the data set identifier MSV000099009 (in-frame mutant data, incl. additional data of mcsB mutant) and MSV000099010 (conditional mutant data).

## 3 Results

### 3.1 Novel *clpX-*deficient strain reveals unexpected expression patterns of the CtsR regulon

**FIGURE 2:**
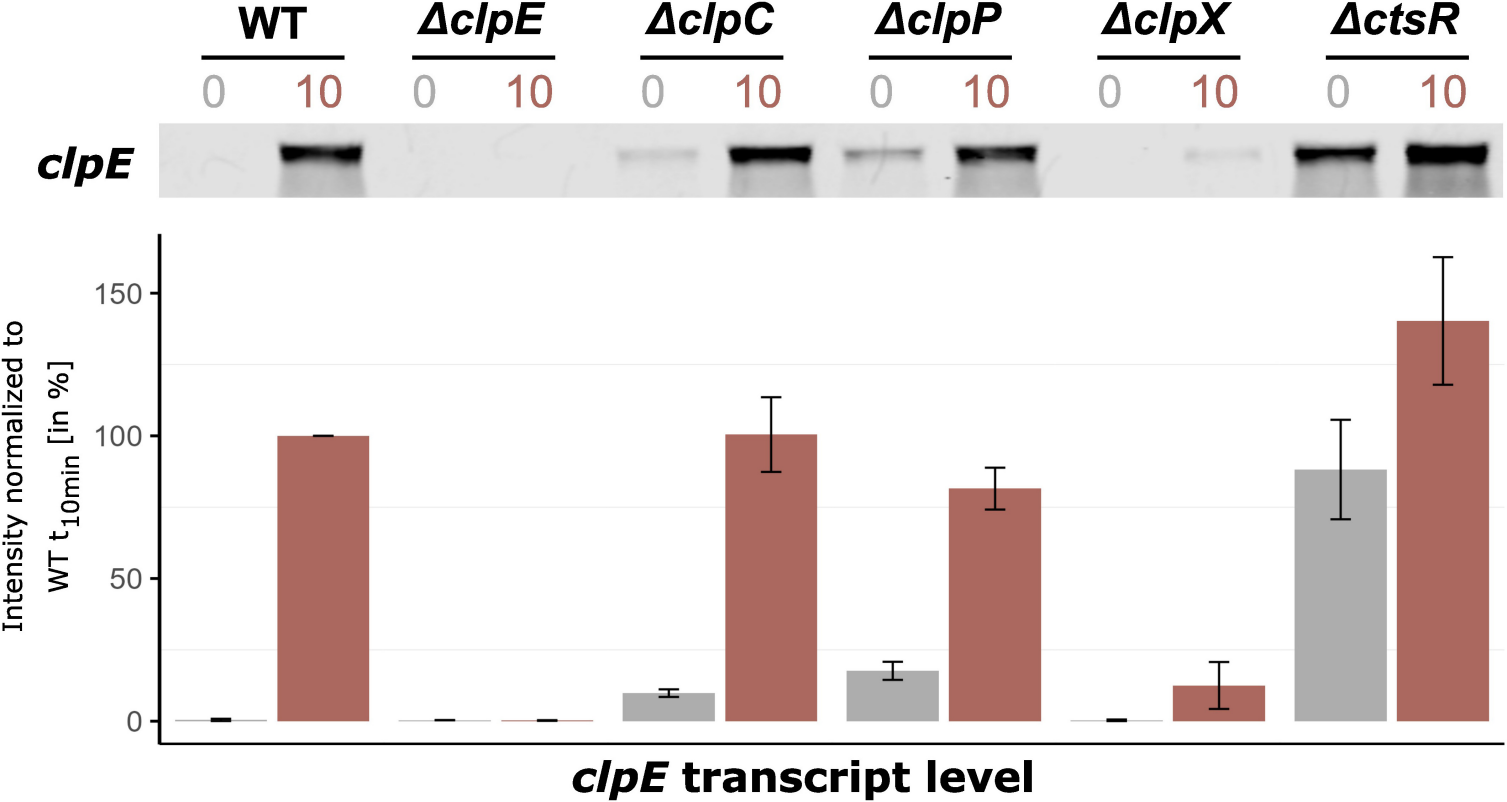
ClpX is involved in derepression of the CtsR regulon under heat stress conditions. Northern blot experiment showing the expression profiles of *clpE*, a gene representative for the CtsR regulon, under control conditions and 10 minutes after heat shock in *B. subtilis* wild-type and its mutants. Cultures were grown in spizizen’s minimal medium with 0.01% yeast extract until the OD_500nm_ of 0.4 (control, t_0_, gray) at 37 °C, then subjected to heat shock in a 50 °C water bath (heat stress, t_10_, red). Below, bar plots display the signal quantification of three independent biological replicates, including their standard deviations. The intensities were normalized to the transcript level of the wild-type 10 min after heat shock set to 100%.

To elucidate the ClpX effect on regulation of the CtsR regulon, the *clpE* gene was selected as the representative marker due to its exclusive regulation by a SigA-type promoter (Derré et al., 1999a), which avoids potential pleiotropic effects associated with SigB. We generated an in-frame *clpX*-deficient strain, which was exposed to heat stress stimulating conditions that activate the CtsR regulon. Using the *clpE* gene as the readout system, we conducted transcriptomic and proteomic profiling with this strain. In agreement with previous studies, no *clpE* transcript was detected either in the *B. subtilis* wild-type (Gerth et al., 2004) or in the novel Δ*clpX* mutant under vegetative growth at 37 °C due to the repression by CtsR (see Figure 2). Heat stress conditions cause a conformational change within the glycin-rich loop in the dimeric CtsR repressor (Elsholz et al., 2010). Ultimately, the dimeric CtsR protein dissociates from its DNA binding and the CtsR regulon is activated. The absence of CtsR already resulted in full induction of *clpE* in the Δ*ctsR* mutant under control conditions. After imposition of heat stress, *clpE* transcript levels increased 1.6-fold resulting in even higher levels compared to the wildtype caused by the activation of the transcriptional regulator Spx. Surprisingly, we observed a decrease in *clpE* transcript levels of approximately 8-fold in the *clpX* mutant strain, when exposed to heat. Given that ClpX is known for its role in protein homeostasis rather than mRNA stability (Gerth et al., 2004, 2008), we hypothesized that ClpX may indirectly modulate the CtsR regulon during heat stress by affecting the stability or dissociation of CtsR from its DNA binding site.

### 3.2 Clp protein network dynamics

**FIGURE 3:**
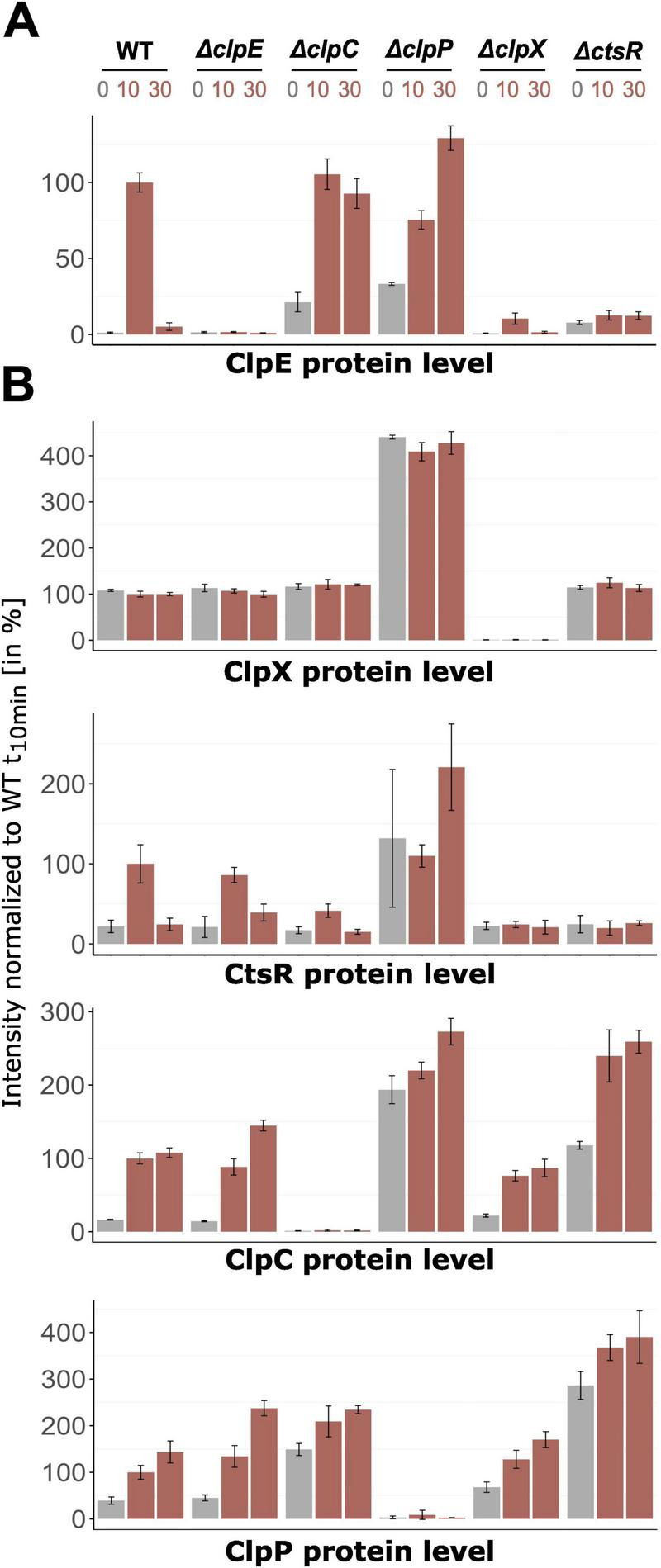
Changes of protein level of the *clpX* gene and CtsR regulon in-frame mutants. Orbitrap Exploris 480 mass spectrometer measurements of **(A)** ClpE protein level and **(B)** CtsR regulon members (ClpC, ClpP, CtsR) and ClpX protein level. Relative abundance in percentage normalized to wild-type t_10_ sample are depicted. X-axis titles indicate plotted protein. DIA-MS analysis was performed with four independent biological replicates in different *B. subtilis* strains and their respective standard deviations are displayed (exceptions see Supplementary Table 6). Control conditions (37 °C) are highlighted in gray, heat shock conditions (10 and 30 min after 50 °C heat shock) in red.

Having shown that ClpX absence impacts the *clpE* transcript level, we extended our investigation to the *clpC* and *clpP* mutants to clarify whether this observed effect was exclusively mediated by ClpX or whether other Clp proteins were involved. Specifically, we aimed to ascertain if the effects on *clpE* transcript levels were mirrored at protein level and to gain a better understanding of the intricate Clp network in response to heat stress. To achieve this, time-resolved mass spectrometry was performed for a comprehensive proteome monitoring, focusing on the levels of ClpE, ClpC, ClpP, ClpX, and CtsR across all previously tested strains (see Figure 3). This experimental data revealed that the protein and *clpE* transcript patterns were consistent across all strains, with the exception of the *ctsR* mutant (see Figures 2, 3A). In this case, the lack of CtsR resulted in notably increased levels of ClpC and ClpP (see Figure 3B). Elevated concentrations of the ClpCP complex mediated degradation of ClpE, which accounts for the lack of ClpE protein accumulation in the *ctsR* mutant upon heat shock (Gerth et al., 2004).

When compared to the *B. subtilis* wild-type, we found that the ClpX-deficient strain showed only slightly increased ClpE levels in response to heat stress, which were 9.6-fold lower (see Figure 3A). This pattern resembled the data obtained for *clpE* on transcript level. Interestingly, the ClpX effect was also extended to CtsR, as the *clpX*-deficient strain possessed lower and non-inducible CtsR levels in comparison to the wild-type (Figure 3B). These findings implied that the presence of ClpX is already essential in the transcriptional derepression of the CtsR regulon. In particular, the appropriate dissociation of CtsR from its DNA-binding motif under heat stress might be compromised in the absence of ClpX.

In the *clpC* mutant, the elevated basal levels of the *clpE* transcript lead to a corresponding increase in ClpE protein levels (see Figures 2, 3A). Derré et al. (2000) postulated a positive role of ClpC on CtsR activity by assisting correct folding and subsequent repression or protecting it from degradation by ClpXP. Wild-type-like CtsR level in the *clpC* mutant (see Figure 3B) supported the notion that lack of ClpC may lead to inactive CtsR, resulting in the observed increase in *clpE* transcript levels under non-inducing conditions. Reduced repression by CtsR impacted not only *clpE* but also other regulon members, such as ClpP (see Figure 3B). Still, the overall regulation remained distinct from what we have seen in the *clpX*-deficient strain. Similarly, while the *clpP* mutant exhibited accumulation of all proteins of interest in this study due to the loss of the essential proteolytic subunit, the transcriptional and proteomic patterns observed in the absence of ClpX were completely different. Thus, the observed particular effect is confined to the lack of ClpX only, highlighting its unique role in modulating the dissociation of CtsR from its DNA binding sites and thereby regulating the broader CtsR regulon under stress conditions.

### 3.3 ClpX affects derepression of the CtsR regulon

**FIGURE 4:**
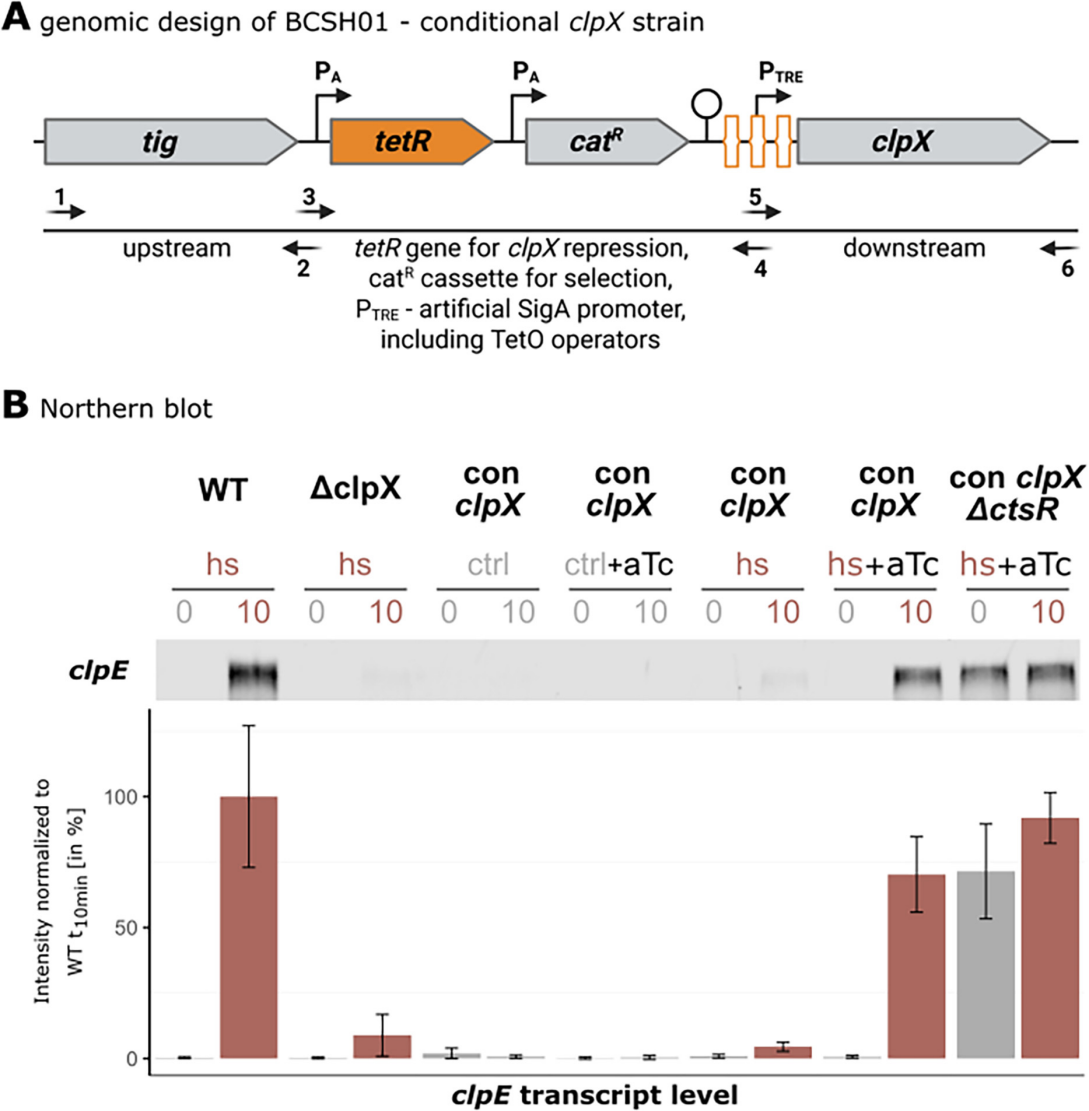
Conditional *clpX* strain construction and Northern blot analysis of *clpE* transcript. **(A)** Detailed genetic construction of the BCSH01 strain. The gene construct containing *tetR*, *cat^R^* and the artificial P_TRE_ promoter was amplified with the primer pair TetR_PsigA-TRE_for and TetR_PsigA-TRE_rev using the chromosomal DNA of BAR610 as template (Harms et al., 2024). Primers used to assemble the transformation construct are listed in Supplementary Table 1. TetR repressor binding sites are schematically highlighted in orange. By introducing a strong terminator after the *tetR*-*cat^R^* fusion, transcriptional initiation of *clpX* in the absence of anhydrotetracycline is prevented. [Created in BioRender. Völker, U. (2025) https://BioRender.com/rkxfmk5]. **(B)** Representative Northern blot analysis of *clpE* transcript levels in a *B. subtilis* wild-type, its isogenic *clpX* mutant and the conditional *clpX* mutant (BCSH01) under various conditions. Application of heat shock (hs, 50 °C in red), vegetative temperatures (ctrl, 37°C in gray) and *clpX* induction 30 min prior to sampling (aTc) is indicated above. Below, bar plots display signal quantification of three independent biological replicates, including their standard deviations normalized to wild-type *clpE* levels 10 min after ongoing heat shock (set as 100%).

In the next step, we generated a conditional mutant (con *clpX*) strain to precisely control the *clpX* expression levels by addition of anhydrotetracycline (aTc), thereby decoupling *clpX* expression from its natural regulatory stimuli (see Figure 4A). This approach allowed us to dissect the direct role of ClpX in real-time, while simultaneously eliminating heat-associated pleiotropic effects. To ensure accurate comparisons, we first titrated the aTc concentration to match the ClpX expression levels observed in *B. subtilis* wild-type (see Supplementary Figure 1). This allowed examination of the ClpX’s effect under finely tuned conditions to directly correlate changes in the expression of the CtsR regulon.

**FIGURE 5:**
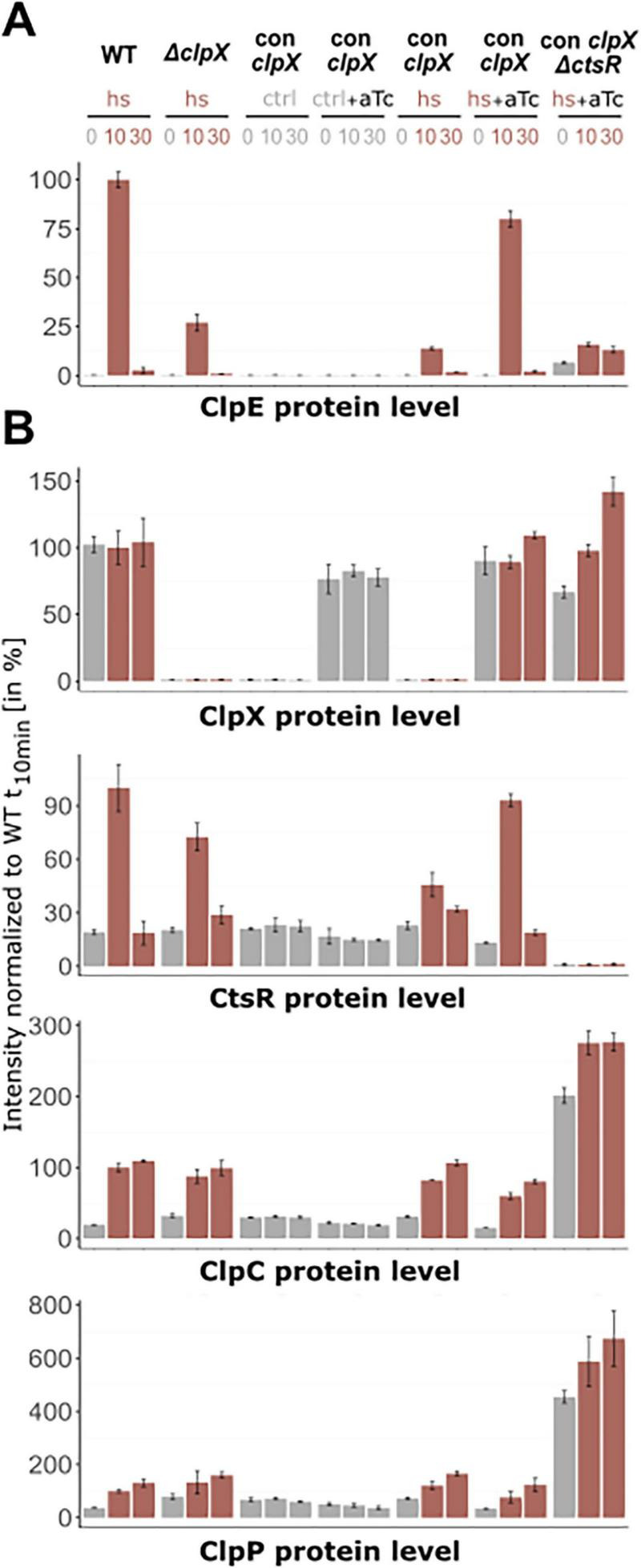
Protein levels in wild-type, Δ*clpX* and conditional *clpX* strains under non-inducing and inducing conditions. Bruker TIMS TOF HT measurement of **(A)** ClpE protein level and **(B)** CtsR regulon members (ClpC, ClpP, CtsR) and ClpX protein level. Relative abundance in percentage normalized to heat stressed wild-type t_10_ sample are depicted. X-axis titles indicate plotted protein. Application of heat shock (hs), vegetative growth (ctrl) and induction (aTc) is indicated above. DIA-MS analysis was performed with three independent biological replicates in different *B. subtilis* strains and their respective standard deviations are displayed (exceptions see Supplementary Table 6). Control conditions (37 °C) are highlighted in grey, heat shock conditions (10 and 30 min after 50 °C heat shock) in red.

Integration of the data of *clpE* transcript and protein levels revealed coherent behavior of the investigated strains (see Figures 4B, 5A). As expected, in the absence of heat, no induction was observed for the *clpE* gene (see Figure 4B) or any corresponding production of ClpE protein (see Figure 5A). Without the heat shock, ClpX alone is insufficient to initiate derepression of the *clpE* gene. This finding underscored that heat is a critical factor required to trigger the conformational shift in CtsR and its concurrent dissociation from the DNA binding site (Elsholz et al., 2010). Notably, the uninduced conditional *clpX* strain exhibited a transcriptional and proteomic profile under heat stress that was almost identical to that of the *clpX* mutant strain. This similarity confirmed that the aTc-induction system effectively mimicked the *clpX* knockout condition, thus validating the conditional *clpX* strain for further investigation of the ClpX-dependent regulatory mechanism. When ClpX was induced to levels comparable to those of the wild-type and heat stress was applied, the transcriptional and proteomic profiles resembled those of the *B. subtilis* wild-type strain with only 1.2-fold difference (see Figures 4B, 5B). When deleting *ctsR* and inducing ClpX production, *clpE* transcription was upregulated even under control (37°C) conditions. However, ClpE protein level displayed similar intensities as in *clpX* deficient strains. This strong discrepancy may be caused by dysregulation and consequently strong upregulation and accumulation of ClpC and ClpP due to the loss of CtsR (see Figure 5B). ClpCP has been reported as the proteolytic complex responsible for the majority of protein degradation (Krüger et al., 2000), likely degrading ClpE to low levels.

## 4 Discussion

In this study, we elucidated the role of ClpX in the regulation of the CtsR regulon in *B. subtilis* under heat stress conditions. Heat and the presence of ClpX are both prerequisites for derepression of the CtsR regulon, a function of ClpX that extends beyond its established role in protein homeostasis. According to current knowledge, CtsR is regarded as a dimeric DNA-binding protein that binds to a conserved heptanucleotide direct repeat sequence (A/GGTCAAA NAN A/GGTCAAA) overlapping with the transcription initiation site or the −35 and −10 boxes of the CtsR regulon promoter and thereby effectively preventing the transcription of its target genes (Derré et al., 1999a,b). CtsR interacts with the major groove of DNA *via* its β-hairpin structure and with the minor groove *via* its helix-turn-helix (HTH) domain (Fuhrmann et al., 2009). The ability of CtsR to sense temperature changes is attributed to its glycine-rich loop which comprises the residues RGGGGY, located at positions G64-G67 adjacent to the HTH domain (Elsholz et al., 2010; Fuhrmann et al., 2009). Our congruent transcript and protein patterns for the *clpE* gene under heat stress conditions demonstrated that ClpX likely performs a crucial chaperone function for the proper dissociation of CtsR. As similar reduction of protein levels of the CtsR regulon was also observed in ClpX-deficient *Staphylococcus aureus* (Busch et al., 2025), this mechanism might be conserved in Gram-positive bacteria.

In contrast to a study conducted by Gerth and coworkers 20 years ago *clpE* mRNA levels in a *clpX* strain indicated very similar expression patterns to a *B. subtilis* wild-type under control (37 °C) as well as heat shock (50 °C) conditions (Gerth et al., 2004). Such variations between studies can potentially originate from different factors:

(i) Retention of the N-terminal zinc finger domain in BEK90: Resequencing of the *clpX*-deficient strain (BEK90) from the previous study revealed that this mutant retains part of a N-terminal zinc finger motif, native *clpX* promoter architecture as well as original protein translation sequences (Shine-Dalgarno sequence and ATG start codon) (see Supplementary Figures 2, 3; Gerth et al., 1996; Gottesman et al., 1993). It has been shown that, in *Escherichia coli*, a zinc finger-like domain of the heat-shock chaperone DnaJ is essential for the recognition and binding to proteins (Szabo et al., 1996). Assuming similar properties for ClpX, it is conceivable that the remaining translated protein still exhibits residual function, which is currently being investigated in further studies. In this study, sequencing of the here constructed in-frame *clpX* mutant strain, as well as congruent data from the uninduced conditional *clpX* strain, verifed the complete lack of ClpX. (ii) Differences in chromosomal background and auxothrophy pattern: Our *clpX* mutant originates from the BSB1 background (Nicolas et al., 2012), whereas the BEK90 derives from the IS58 (*trpC2 lys-3*) (Gerth et al., 2004), which is auxotrophic for both tryptophan and lysine. Auxotrophy can influence cellular physiology, particularly under stress conditions. Under heat stress, *B. subtillis* requires high amounts of energy as well as amino acid precursors for refolding and resynthesizing of damaged proteins, while activity of transport proteins and proteins involved in amino acid uptake are impaired. In auxotrophic strains such as BEK90, limitations in amino acid supply can cause altered expression profiles (including *clpE* expression) compared to the prototroph *clpX* mutant used in this study.

**FIGURE 6:**
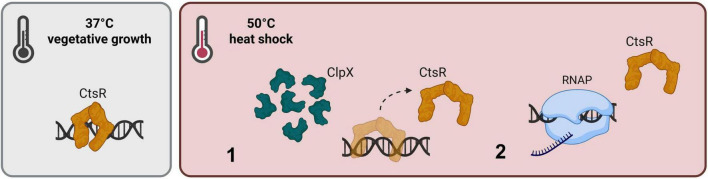
Adjusted model for heat- and ClpX-dependent regulation of the CtsR activity. CtsR operates as a dimeric DNA repressor under control conditions and binds to its conserved heptanucleotide sequence, preventing transcription of the CtsR regulon. (1) A sudden increase in temperature, results in a heat-induced conformational change of CtsR, mediated by the chaperone function of ClpX. Thus, transcriptional initiation of regulated genes is activated (2). [Created in BioRender. Völker, U. (2025) https://BioRender.com/6gz6yh3].

Our data indicate that upon heat stress conditions, ClpX seems to influence the heat-induced conformational change of CtsR and the induction of the CtsR regulon. The chaperone function of ClpX may be involved in stabilizing an intermediate conformation of CtsR or directly assisting in the refolding of CtsR into a non-DNA-binding-state. Experimental data from Δ*clpC* and Δ*clpP* mutants have shown that this phenomenon is solely attributed to ClpX (see Figure 3). Based on these new insights, we propose an adjusted revised model of CtsR regulation that builds on the work of Elsholz and coworkers (see Figure 6; Elsholz et al., 2010).

Class three stress gene repressor represses its regulon by binding to its conserved DNA binding motif under vegetative growth conditions. Simultaneously, McsB is kept inactive by the complex formation with ClpC (Kirstein et al., 2005). Heat exposure results in a conformational change within the winged HTH-domain of CtsR, a process likely supported by the chaperone function of ClpX. This conformational state of CtsR loses its DNA binding affinity and detaches from its DNA binding site, while the transcription of the CtsR regulon is initiated. Non-functional CtsR is targeted for phosphorylation by the heat-activated McsB-P/McsA complex and thereby tagged for ClpCP-dependent degradation (Elsholz et al., 2011).

The current model does not clearly describe how CtsR is reactivated as a DNA-binding repressor under heat stress conditions. According to the model, CtsR reactivation occurs despite ongoing heat stress and the potential heat-induced conformational change of newly synthesized CtsR. We suggest that the availability of ClpX during heat stress plays a pivotal role in the feedback loop of the CtsR regulon. Our data show that ClpX protein levels under heat stress were identical to those observed during vegetative growth. A study by Kirstein et al. (2008) revealed that Clp-associated proteins primarily localize in clusters at the cell poles or mid-cell region under heat stress to recycle damaged proteins. In case, a significant portion of ClpX is sequestered in a complex with ClpP, it may no longer support the conformational shift required for CtsR to effectively dissociate from DNA. Thus, we assume ClpX availability is critical for maintaining the balance of CtsR activity and for regulating CtsR-dependent genes under heat-stress conditions.

## Data Availability

The datasets presented in this study can be found in online repositories. The names of the repository/repositories and accession number(s) can be found in the article.
