# Supplementary material for: Requirement of ClpX for CtsR dissociation from its operator elements upon heat stress in Bacillus subtilis
Source: Front Microbiol. 2025 Nov 7;16:1699655. doi: 10.3389/fmicb.2025.1699655 (PMC12634643; doi:10.3389/fmicb.2025.1699655)
Supplement: Supplementary file 1 [file Supplementary_file_1.zip › Suppementary Figures (S1-S3 and Tables (S1-S6).docx]

**
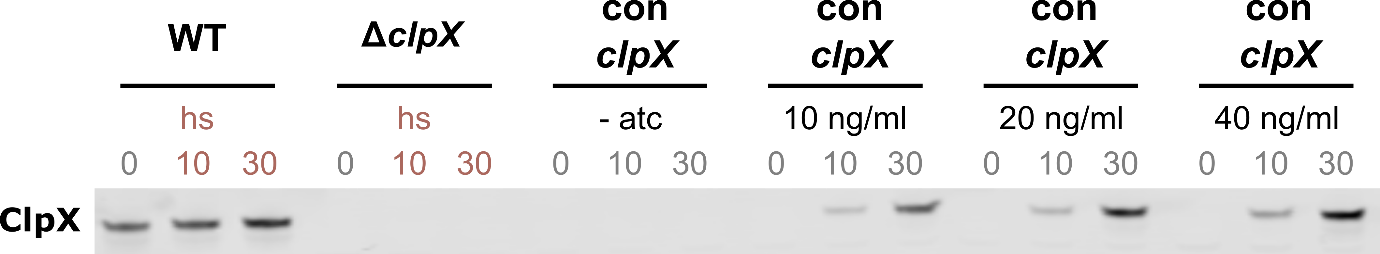
**

**Supplementary Figure S1: Determination of optimal aTc concentration used for conditional *clpX* induction obtaining wild-type protein levels.**

Western blot analysis ClpX protein levels in a *B. subtilis* wild-type, its isogenic *clpX* mutant and the conditional *clpX* mutant (con *clpX* = BCSH01) under various conditions: 10 min and 30 min after heat shock (50°, highlighted in red) and the addition of 0, 10, 20 or 40 ng/ml anhydrotetracycline 30 min prior to the sampling (atc). Grey indicates growth at 37°C. Based on this experimental result, the addition of 20 ng/ml aTc 30 min prior to the sampling was chosen as the concentration for the induction of the *clpX* gene to ensure ClpX levels comparable to that of the wild-type.

**
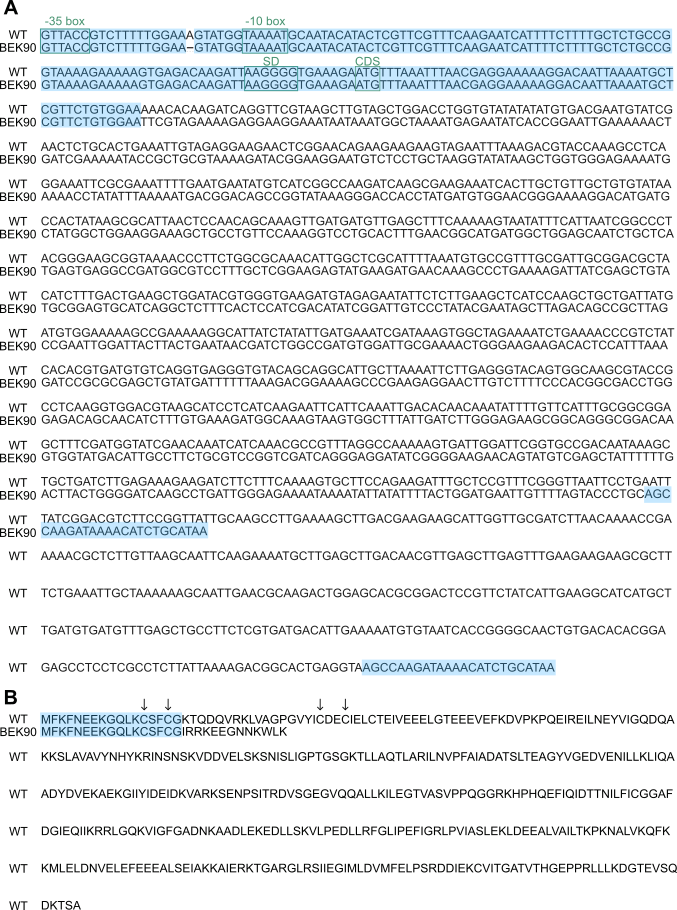
**

**Supplementary Figure S2: BEK90 *clpX* strain contains remaining truncated ClpX**

(**A**) DNA and (**B**) protein alignment of a *B. subtilis* wild-type (WT) and the *clpX* mutant (BEK90) from Gerth *et al.* (1). Blue highlighted sequences display identity between the wild-type and the BEK90 strain. The -35 and -10 boxes correspond to the SigA promoter of the *clpX* gene, SD represents the Shine Dalgarno and CDS the start of the coding sequence. Within the protein alignment of the native ClpX and the residual N-terminal ClpX of BEK90 the first 17 residues are identical. BEK90 ClpX has a length of 30 residues, arrows indicate cysteine residues of the zinc-finger motif.

**
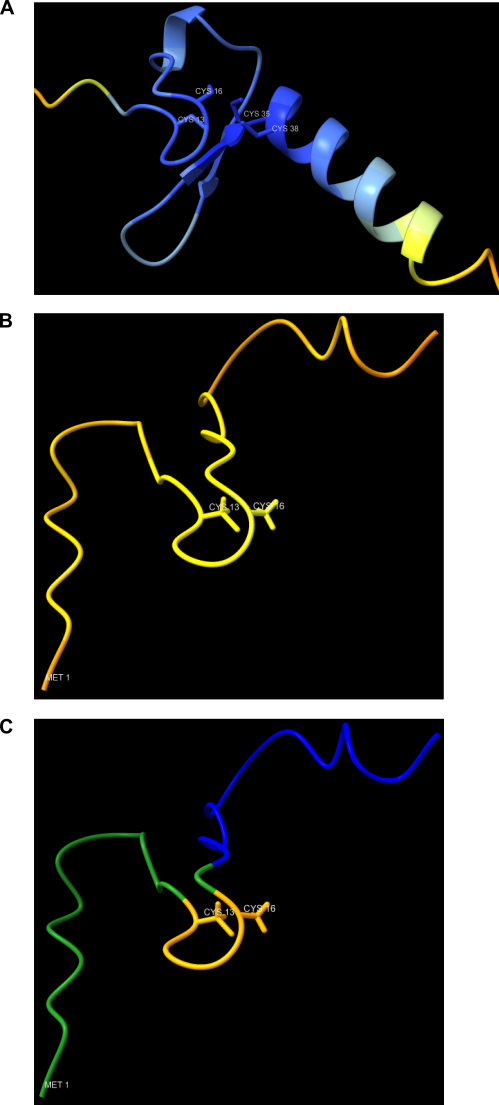
**

**Supplementary Figure S3: Structure prediction of truncated BEK90 ClpX**

Structure prediction using AlphaFold (2) and ChimeraX (3). (**A**) Structure of the N-terminus of wildtype ClpX, derived from UniProt (P50866). Four cysteine residues, responsible for zinc ion coordination are labeled. Color code of structure resembles model prediction confidence according to Jumper *et al.* (2) (dark blue - very high, light blue – high, yellow – low, orange – very low). (**B**) and (**C**) structure of truncated ClpX from BEK90 strain. Two remaining cysteine residues are labeled. Color code of structure resembles model prediction confidence according to Jumper *et al.* (2) in (**B**) or conserved residues (green and yellow), part of zinc-finger domain (yellow) and unconserved residues (blue) in (**C**).

**Supplementary table S1.** Primers used for strain generation and NIR Northern blot probes

| **Purpose** | **Name** | **Sequence (5’-3’)** |
| --- | --- | --- |
| BZZ01 | DclpC_up_for  DclpC_up_rev  DclpC_do_for  DclpC_do_rev | CCGGTGATATTCTCATTTTAGTCATATCGATTCATCCTCCTG  CCGGTGATATTCTCATTTTAGTCATATCGATTCATCCTCCTG  CATCTTACTCGATGAACTCTTCTAATATAGAAGACGGAAATGAGGC  CTATTCCCGTTGCCATACGCC |
| BZZ02 | DclpE_up_for  DclpE_up_rev  Res_SigA_Phleo_for  Res_Phleo_just_rev  DclpE_do_for  DclpE_do_rev | CTCGTAATATAAGCCGTGATC  ATATAAGCAATCAGTCATGTCAAGTCTTATGCACAAAAATTTTTG  TTGACATGACTGATTGCTTATATTATAATGTCAAAGTAACACGAAGGAGGAGGG  TAAGATGTTACAGTCTATCCCGGC  TTAGCTCTTGATCTGTTGGAAG  CTTCCAACAGATCAAGAGCTAACAAGCTGCTAATTTCAGTAGACC  GAGAAGAGTAAGGATGTCGG |
| BZZ03 | DclpP_up_for  DclpP_up_rev  Res_ Spec_for  Res_Spec_rev  DclpP_do_for  DclpP_do_rev | TCCATCGGAACAGGTGAAGC  CATAATGCTCCTCCTTCACC  GGTGAAGGAGGAGCATTATGAACACGTACGAGCAGATC  GTCAATCAGGCCGTATTCAAGTTACAACTTCTTTAAGCGGTTGTTC  CTTGAATACGGCCTGATTGAC  CTGTTGCAGGAGAATGATCC |
| BZZ04 | DclpX_up_for  DclpX_up_rev  Res_clpX_Ery_for  Res_clpX_Ery_rev  DclpX_do_for  DclpX_do_rev | CATCCCTGGCTTCGAAGATC  TTTGTTCATTCTTTCACCCCTTAATCTTGTCTCACTTTTTCTTTTACCGG  GGGGTGAAAGAATGAACAAAAATATAAAATATTCTCAAAAC  TTATTTCCTCCCGTTAAATAATAG  CTATTATTTAACGGGAGGAAATAAAGATAAGCACAAACCTCCTGAG  CAACCGTTTTCAGCTCGTCC |
| BZZ05 | DctsR_up_for  DctsR_up_rev  Res_Km_just_for  Res_Km_just_rev  DctsR_do_for  DctsR_do_rev | CATTCGCAGAGTGTAAAGGC  GGTGATATTCTCATTTTAGTCATTCAACCCCCTCCTTTACTG  ATGACTAAAATGAGAATATCACCGG  GAAGAGTTCATCGAGTAAGATGTAGTACTTGATTTTCTCCCAATCAGGCTTGATCC  CTTACTCGATGAACTCTTCTAAGCGGGTGAAAAGATTGATTTG  CTTCAATCCAGTCGTCGACC |
| BCSH01 | 1_cond_ClpX_up_for  2_cond_ClpX_up_rev  3_TetR_PsigA-TRE_for  4_TetR_PsigA-TRE_rev  5_cond_ClpX_do_for  6_cond_ClpX_do_rev | AATCGAGCCTGTAGACCGTCCTG  ACGTTTGCGTGCCAATTCGTTTCTGACTTGCACATTCTATATG  GAATTGGCACGCAAACGTAAAC  AAGGATCCCTATCACTGATAGGGAATCTATCTTAATTATATC  ATCAGTGATAGGGATCCTTTACTCGTTCGTTTCAAGAATC  CTCCTGAGTGTTACCACTCAGGAG |
| *clpE* probe | clpE_Nor_for  clpE_T7_rev | GCATCTCTTGTTGCAAACAC  GAAATTAATACGACTCACTATAGGGAGAATGATAAAGTGACACATGCTTTGATTG |

**Supplementary table S2.** Parameter for mass spectrometric analysis using an Orbitrap Exploris™ 480 mass spectrometer (Thermo Fisher Scientific) coupled to an UltiMate™ 3000 RSLC nano system (Thermo Fisher Scientific)

| Reversed phase liquid chromatography (RPLC) | |
| --- | --- |
| Instrument | Ultimate 3000 RSLC (Thermo Fisher Scientific) |
| Trap column | 75 μm inner diameter, packed with 3 μm C18 particles (Acclaim PepMap100, Thermo Scientific) |
| Analytical column | Accucore 150-C18, (Thermo Fisher Scientific)  25 cm x 75 μm, 2,6 μm C18 particles, 150 Å pore size |
| Buffer system | binary buffer system consisting of 0.1% acetic acid in HPLC-grade water (buffer A) and 100% ACN in 0.1% acetic acid (buffer B) |
| Flow rate | 300 nl/min |
| Gradient | linear gradient of buffer B from 2% up to 25% |
| Gradient duration | 60 min |
| Column oven temperature | 40°C |
| **mass spectrometry** | |
| instrument | Orbitrap Exploris™ 480 mass spectrometer (Thermo Fisher Scientific) |
| Electrospray | Nanospray Flex Ion Source |
| Operation mode | data-independent |
| **Full MS** |  |
| MS scan resolution | 120000 |
| Norm. AGC target (%) | 300 |
| maximum ion injection time for the MS scan | 60 ms |
| Scan range | 350 to 1200 m/z |
| RF Lens | 50 % |
| Spectra data type | profile |
| **dd-MS2** |  |
| Precursor mass range | 350 to 1200 m/z |
| Resolution | 30,000 |
| Norm. MS/MS AGC target (%) | 3000 |
| Maximum ion injection time mode | auto |
| Spectra data type | profile |
| Microscans | 1 |
| Isolation window | 66 windows, 13 m/z, 2 m/z overlap |
| Define first mass | 200 |
| Dissociation mode | higher energy collisional dissociation (HCD) |
| HCD normalized collision energy | 30% |

**Supplementary table S3.** *Parameter for mass spectrometric analysis using Bruker TIMS TOF HT* *(Bruker Daltonics GmbH) coupled to an Evosep One System (Evosep Biosystems Aps)*

| **Nano RPLC** | |
| --- | --- |
| Column system | Evosep One (Evosep Biosystems Aps)  Evotip Pure, sample loaded by following guide given by Evosep  Evosep Performance column EV-1137, 15cm x 150um, 1.5um C18 column heated to 40degC  30SPD LC method by Evosep |
| Buffer system | binary buffer system consisting of 0.1% formic acid in HPLC-grade water (buffer A) and 100% ACN in 0.1% formic acid (buffer B) |
| **mass spectrometry** | |
| Electrospray | Captive spray 2 Emitter 10 um part no, 1811112, Spraying Voltage: 1700v |
| Operation mode | data-independent |
| MS and MS/MS scan range | 100-1700m/z positive polarity data acquired with dia-PASEF mode activated in 400-1001 m/z, 30 m/z windows and 1.17 sec cycle time |
| mobility range | 0.65-1.45 V-s/cm2 |
| ramp and accumulation time | 100ms |
| collision energy ramping | for mobility range 0.6-1.60 V-s/cm2 was 20-59eV |

**Supplementary table S4.** *Detailed search parameters of the in-frame mutant DIA-MS data set in Spectronaut Version 16*

Spectronaut 16.3.221108.53000

Computer Name: AGVOE-SPECTRONA

User Domain Name: AGVOE-SPECTRONA

User Name: spectronaut

Analysis Mode: UI

Analysis Type: directDIA

[BEGIN-SETTINGS]

Settings Used: C_FunGene_directDIA_sparse_no_imputing

├─ DIA Analysis\Calibration

│ ├─ MZ Extraction Strategy: Maximum Intensity

│ ├─ Allow source specific iRT Calibration: True

│ ├─ Precision iRT: True

│ │ ├─ Exclude De-amidated Peptides: True

│ │ └─ iRT <-> RT Regression Type: Local (Non-Linear) Regression

│ ├─ MS1 Mass Tolerance Strategy: System Default

│ └─ MS2 Mass Tolerance Strategy: System Default

├─ DIA Analysis\Identification

│ ├─ Precursor Qvalue Cutoff: 0.001

│ ├─ Precursor PEP Cutoff: 0.2

│ ├─ Protein Qvalue Cutoff (Experiment): 0.01

│ ├─ Protein Qvalue Cutoff (Run): 0.05

│ ├─ Single Hit Definition: By Stripped Sequence

│ ├─ Exclude Single Hit Proteins: False

│ ├─ Exclude Duplicate Assays: True

│ ├─ Exclude Predicted Fragment Scores: False

│ ├─ Generate Decoys: True

│ │ ├─ Decoy Generation Method: Mutated

│ │ │ └─ Preferred Fragment Source: NN Predicted Fragments

│ │ └─ Decoy Limit Strategy: Dynamic

│ │ └─ Library Size Fraction: 0.1

│ └─ Pvalue Estimator: Kernel Density Estimator

├─ DIA Analysis\Pipeline Mode

│ ├─ Generate SNE File: True

│ │ └─ Store Ion traces in SNE: False

│ ├─ Post Analysis Reports:

│ │ ├─ CV Density Line Chart: True

│ │ ├─ CVs Below X Bar Chart: True

│ │ ├─ Data Completeness Bar Chart: True

│ │ ├─ Run Identifications Bar Chart: True

│ │ └─ Scoring Histograms: True

│ ├─ Report Schema: C_FunGene_complex (Normal)

│ └─ Reporting Unit: Across Experiment

├─ DIA Analysis\Post Analysis

│ ├─ Differential Abundance Testing: Paired t-test

│ │ └─ Group-Wise Testing Correction: False

│ ├─ Differential Abundance Grouping: Major Group (Quantification Settings)

│ │ └─ Smallest Quantitative Unit: Precursor Ion (Quantification Settings)

│ │ └─ Use All MS-Level Quantities: False

│ ├─ Calculate Explained TIC: None

│ ├─ Calculate Sample Correlation Matrix: True

│ └─ Hierarchical Clustering: True

│ ├─ Distance Metric: Manhattan Distance

│ ├─ Linkage Strategy: Ward's Method

│ ├─ Order Runs by Clustering: True

│ └─ Z-score Transformation: False

├─ DIA Analysis\Protein Inference

│ └─ Protein Inference Workflow: Automatic

│ └─ Inference Algorithm: IDPicker

├─ DIA Analysis\PTM Workflow

│ └─ PTM Localization: True

│ ├─ Probability Cutoff: 0.75

│ └─ PTM Analysis: True

│ ├─ Hierarchical Clustering: False

│ ├─ Multiplicity: True

│ ├─ Flanking Region: 7

│ └─ PTM Consolidation: Sum

├─ DIA Analysis\Quantification

│ ├─ Precursor Filtering: Identified (Qvalue)

│ │ └─ Imputation Strategy: Use Background Signal

│ ├─ Proteotypicity Filter: None

│ ├─ Protein LFQ Method: Automatic

│ ├─ Quantity MS Level: MS2

│ ├─ Quantity Type: Area

│ ├─ Cross-Run Normalization: True

│ │ ├─ Normalization Filter Type: None

│ │ ├─ Normalization Strategy: Local Normalization

│ │ └─ Row Selection: Identified in at least 1 Run (Sparse)

│ ├─ Interference Correction: True

│ │ ├─ Only Identified Peptides: True

│ │ ├─ Exclude All Multi-Channel Interferences: True

│ │ ├─ MS1 Min: 2

│ │ └─ MS2 Min: 3

│ ├─ Major (Protein) Grouping: by Protein Group Id

│ ├─ Minor (Peptide) Grouping: by Stripped Sequence

│ ├─ Major Group Quantity: Mean peptide quantity

│ ├─ Major Group Top N: True

│ │ ├─ Max: 3

│ │ └─ Min: 2

│ ├─ Minor Group Quantity: Sum precursor quantity

│ └─ Minor Group Top N: False

├─ DIA Analysis\Workflow

│ ├─ Method Evaluation: False

│ ├─ MS2 DeMultiplexing: Automatic

│ ├─ Profiling Strategy: iRT Profiling

│ │ ├─ Carry-over exact Peak Boundaries: False

│ │ ├─ Profiling Row Selection: Minimum Qvalue Row Selection

│ │ │ └─ Qvalue Threshold: 0.001

│ │ └─ Profiling Target Selection: Profile only non-identified Precursors

│ │ ├─ Identification Criterion: Qvalue

│ │ └─ Threshold: 0.001

│ ├─ Run Limit for directDIA Library: -1

│ └─ Unify Peptide Peaks Strategy: Select corresponding Peak

├─ DIA Analysis\XIC Extraction

│ ├─ XIC IM Extraction Window: Dynamic

│ │ └─ Correction Factor: 1

│ ├─ XIC RT Extraction Window: Dynamic

│ │ └─ Correction Factor: 1

│ ├─ MS1 Mass Tolerance Strategy: Dynamic

│ │ └─ Correction Factor: 1

│ └─ MS2 Mass Tolerance Strategy: Dynamic

│ └─ Correction Factor: 1

├─ Pulsar Search\Identification

│ ├─ PSM FDR: 0.01

│ ├─ Peptide FDR: 0.01

│ ├─ Protein Group FDR: 0.01

│ └─ PTM Localization Filter: False

├─ Pulsar Search\Labeling

│ └─ Channels:

│ ├─ Channel 1: False

│ ├─ Channel 2: False

│ └─ Channel 3: False

├─ Pulsar Search\Modifications

│ ├─ Max Variable Modifications: 5

│ └─ Select Modifications:

│ ├─ Fixed Modifications::

│ └─ Variable Modifications: : Oxidation (M)

├─ Pulsar Search\Peptides

│ ├─ Enzymes / Cleavage Rules: Trypsin/P, LysC

│ ├─ Digest Type: Specific

│ ├─ Max Peptide Length: 52

│ ├─ Min Peptide Length: 7

│ ├─ Missed Cleavages: 2

│ └─ Toggle N-terminal M: True

├─ Pulsar Search\Result Filters

│ ├─ Fragment Ions:

│ │ ├─ Ion AA Length: True

│ │ │ └─ N: 3

│ │ ├─ Ion Charge: False

│ │ ├─ Ion Loss Type: False

│ │ ├─ Ion Type: False

│ │ ├─ m/z : True

│ │ │ ├─ Max: 1800

│ │ │ └─ Min: 300

│ │ └─ Relative Intensity: True

│ │ └─ Min: 5

│ └─ Precursors:

│ ├─ Amino Acids: False

│ ├─ Best N Fragments per Peptide: True

│ │ ├─ Max: 10

│ │ └─ Min: 6

│ ├─ Best N Peptides per Protein Group: False

│ ├─ Channel Count: False

│ ├─ FASTA Matched: False

│ ├─ Missed Cleavage: False

│ ├─ Modifications: None

│ ├─ Peptide Charge: False

│ └─ Proteotypicity: False

├─ Pulsar Search\Speed-Up

│ └─ MS2 Index: Automatic

**Supplementary table S5.** *Detailed search parameters of the conditional mutant DIA-MS data set in Spectronaut Version 20*

Spectronaut 20.0.250606.92449

Computer Name: AGVOE-HECHT

User Domain Name: AGVOE-HECHT

User Name: Spectronaut

Analysis Mode: UI

Analysis Type: directDIA

Analysis Date: 02-July-2025 17:52:02 UTC +02:00

[BEGIN-SETTINGS]

Settings Used: C_FunGene_directDIA_sparse_no_imputing

├─ DIA Analysis\Calibration

│ ├─ MZ Extraction Strategy: Maximum Intensity

│ ├─ Allow source specific iRT Calibration: True

│ ├─ Precision iRT: True

│ │ ├─ Exclude De-amidated Peptides: True

│ │ └─ iRT <-> RT Regression Type: Local (Non-Linear) Regression

│ ├─ MS1 Mass Tolerance Strategy: System Default

│ └─ MS2 Mass Tolerance Strategy: System Default

├─ DIA Analysis\Identification

│ ├─ Precursor Qvalue Cutoff: 0.001

│ ├─ Precursor Qvalue Cutoff (Experiment): 0.01

│ ├─ Precursor PEP Cutoff: 0.2

│ ├─ Protein Qvalue Cutoff (Experiment): 0.01

│ ├─ Protein Qvalue Cutoff (Run): 0.05

│ ├─ Protein PEP Cutoff: 0.75

│ ├─ Single Hit Definition: By Stripped Sequence

│ ├─ Single Hit Protein Rule: Stratified Single Hit Protein FDR

│ ├─ Run-Level Protein Scoring: Highest Scoring Observation (SN19 default)

│ ├─ Exclude Duplicate Assays: True

│ ├─ Exclude Predicted Fragment Scores: False

│ ├─ Generate Decoys: True

│ │ ├─ Decoy Generation Method: Mutated

│ │ │ └─ Preferred Fragment Source: NN Predicted Fragments

│ │ └─ Decoy Limit Strategy: Dynamic

│ │ └─ Library Size Fraction: 0.1

│ └─ Pvalue Estimator: Kernel Density Estimator

├─ DIA Analysis\Pipeline Mode

│ ├─ Export All XICs: False

│ ├─ Generate SNE File: True

│ │ └─ Store Ion traces in SNE: False

│ ├─ Post Analysis Reports:

│ │ ├─ Binned CVs: False

│ │ ├─ Binned Identification: False

│ │ ├─ CV Density Line Chart: True

│ │ ├─ CVs Below X Bar Chart: True

│ │ ├─ Data Completeness Bar Chart: True

│ │ ├─ Run Identifications Bar Chart: True

│ │ ├─ Scoring Histograms: True

│ │ └─ TIC Overlay: False

│ ├─ PTM Report Schema:

│ ├─ Report Schema: SpectroPipeR (Normal)

│ └─ Reporting Unit: Across Experiment

├─ DIA Analysis\Post Analysis

│ ├─ Differential Abundance Testing: Paired t-test

│ │ ├─ Group-Wise Testing Correction: False

│ │ ├─ Log2 Ratio Candidate Filter: 0.58

│ │ └─ Confidence Candidate Filter: Qvalue

│ │ └─ Confidence: 0.05

│ ├─ Differential Abundance Grouping: Major Group (Quantification Settings)

│ │ └─ Smallest Quantitative Unit: Precursor Ion (Quantification Settings)

│ │ └─ Use All MS-Level Quantities: False

│ ├─ Calculate Explained TIC: Quick

│ ├─ Calculate Sample Correlation Matrix: True

│ ├─ Gene Ontology: C:\Users\Spectronaut\AppData\Roaming\Spectronaut\geneOntology\Ontologies\bgs_default_go-basic.obo

│ └─ Hierarchical Clustering: True

│ ├─ Distance Metric: Manhattan Distance

│ ├─ Linkage Strategy: Ward's Method

│ ├─ Order Runs by Clustering: True

│ └─ Z-score Transformation: False

├─ DIA Analysis\Protein Inference

│ └─ Protein Inference Workflow: Automatic

│ └─ Inference Algorithm: IDPicker

├─ DIA Analysis\PTM Workflow

│ ├─ Input Normalization Strategy: None

│ └─ PTM Localization: False

├─ DIA Analysis\Quantification

│ ├─ Precursor Filtering: Identified (Qvalue)

│ │ ├─ Imputation Strategy: Use Background Signal

│ │ └─ Multi Channel Qvalue Filter: Group Qvalue

│ ├─ Proteotypicity Filter: None

│ ├─ Protein LFQ Method: MaxLFQ

│ ├─ Quantity MS Level: MS2

│ ├─ Quantity Type: Area

│ ├─ Cross-Run Normalization: True

│ │ ├─ Normalization Filter Type: None

│ │ ├─ Normalization Strategy: Global Normalization

│ │ │ └─ Normalize on: Median

│ │ └─ Row Selection: Identified in at least 1 Run (Sparse)

│ ├─ Perform background noise removal: True

│ ├─ Quantification window: Not Synchronized (SN 17)

│ ├─ Interference Correction: True

│ │ ├─ Only Identified Peptides: True

│ │ ├─ Exclude All Multi-Channel Interferences: True

│ │ ├─ MS1 Min: 2

│ │ └─ MS2 Min: 3

│ ├─ Major Group Quantity: Mean peptide quantity

│ ├─ Minor (Peptide) Grouping: by Stripped Sequence

│ ├─ Major (Protein) Grouping: by Protein Group Id

│ ├─ Major Group Top N: True

│ │ ├─ Max: 3

│ │ └─ Min: 2

│ ├─ Minor Group Quantity: Sum precursor quantity

│ ├─ Minor Group Top N: False

│ ├─ Use Log2 Quantity Filter: False

│ └─ Perform IM Peak Picking for Quantification: True

├─ DIA Analysis\Workflow

│ ├─ Method Evaluation: False

│ ├─ MS2 DeMultiplexing: Automatic

│ ├─ Multi-Channel Workflow Definition: From Library Annotation

│ │ └─ Fallback Option: Labeled

│ ├─ Profiling Strategy: iRT Profiling

│ │ ├─ Carry-over exact Peak Boundaries: False

│ │ ├─ Profiling Row Selection: Minimum Qvalue Row Selection

│ │ │ └─ Qvalue Threshold: 0.001

│ │ └─ Profiling Target Selection: Profile only non-identified Precursors

│ │ ├─ Identification Criterion: Qvalue

│ │ └─ Threshold: 0.001

│ ├─ Run Limit for directDIA Library: -1

│ ├─ Hybrid (DDA + DIA) Library: False

│ └─ Unify Peptide Peaks Strategy: Select corresponding Peak

├─ DIA Analysis\XIC Extraction

│ ├─ XIC IM Extraction Window: Dynamic

│ │ └─ Correction Factor: 1

│ ├─ XIC RT Extraction Window: Dynamic

│ │ └─ Correction Factor: 1

│ ├─ MS1 Mass Tolerance Strategy: Dynamic

│ │ └─ Correction Factor: 1

│ └─ MS2 Mass Tolerance Strategy: Dynamic

│ └─ Correction Factor: 1

├─ Pulsar Search\Identification

│ ├─ PSM FDR: 0.01

│ ├─ Peptide FDR: 0.01

│ ├─ Protein Group FDR: 0.01

│ ├─ directDIA Workflow: directDIA+ (Deep)

│ └─ PTM Localization Filter: False

├─ Pulsar Search\iRT Calibration

│ ├─ Calibrate from Empirical RT: False

│ ├─ Auto-assign iRT source: True

│ ├─ iRT Reference Strategy: Deep Learning Assisted iRT Regression

│ ├─ Use Source Specific iRT: Auto

│ └─ Minimum Rsquare: 0.8

├─ Pulsar Search\Labeling

│ └─ Channels:

│ ├─ Channel 1: False

│ ├─ Channel 2: False

│ ├─ Channel 3: False

│ ├─ Channel 4: False

│ └─ Channel 5: False

├─ Pulsar Search\Modifications

│ └─ Search Mode: Closed Search

│ ├─ Max Variable Modifications: 5

│ ├─ Fixed Modifications::

│ └─ Variable Modifications:: Oxidation (M)

├─ Pulsar Search\Peptides

│ ├─ Enzymes / Cleavage Rules: Trypsin/P

│ ├─ Digest Type: Specific

│ ├─ Decoy Generation Rule: KR

│ ├─ Max Peptide Length: 52

│ ├─ Min Peptide Length: 7

│ ├─ Missed Cleavages: 2

│ └─ Toggle N-terminal M: True

├─ Pulsar Search\Result Filters

│ ├─ Fragment Ions:

│ │ ├─ Ion AA Length: True

│ │ │ └─ N: 3

│ │ ├─ Ion Charge: False

│ │ ├─ Ion Loss Type: False

│ │ ├─ Ion Type: False

│ │ ├─ m/z : True

│ │ │ ├─ Max: 1800

│ │ │ └─ Min: 300

│ │ ├─ Overlapping between Channels: False

│ │ └─ Relative Intensity: True

│ │ └─ Min: 5

│ └─ Precursors:

│ ├─ Amino Acids: False

│ ├─ Best N Fragments per Peptide: True

│ │ ├─ Max: 10

│ │ └─ Min: 6

│ ├─ Best N Peptides per Protein Group: False

│ ├─ Channel Count: False

│ ├─ FASTA Matched: False

│ ├─ Missed Cleavage: False

│ ├─ Modifications: None

│ ├─ Peptide Charge: False

│ └─ Proteotypicity: False

├─ Pulsar Search\Speed-Up

│ ├─ MS2 Index: Automatic

│ └─ diaPASEF Pre-Processing: Automatic

├─ Pulsar Search\Tolerances

│ └─ Tolerance Parameters:

│ ├─ Thermo IonTrap:

│ │ ├─ Calibration Search: Dynamic

│ │ │ ├─ MS1 Correction Factor: 1

│ │ │ └─ MS2 Correction Factor: 1

│ │ └─ Main Search: Dynamic

│ │ ├─ MS1 Correction Factor: 1

│ │ └─ MS2 Correction Factor: 1

│ ├─ Thermo Orbitrap:

│ │ ├─ Calibration Search: Dynamic

│ │ │ ├─ MS1 Correction Factor: 1

│ │ │ └─ MS2 Correction Factor: 1

│ │ └─ Main Search: Dynamic

│ │ ├─ MS1 Correction Factor: 1

│ │ └─ MS2 Correction Factor: 1

│ └─ TOF:

│ ├─ Calibration Search: Dynamic

│ │ ├─ MS1 Correction Factor: 1

│ │ └─ MS2 Correction Factor: 1

│ └─ Main Search: Dynamic

│ ├─ MS1 Correction Factor: 1

│ └─ MS2 Correction Factor: 1

└─ Pulsar Search\Workflow

├─ Fragment Ion Selection Strategy: Intensity Based

├─ In-Silico Generate Missing Channels: False

└─ Use DNN Predicted Ion Mobility: Auto

[END-SETTINGS]

**Supplementary table S6.** *Samples removed manually from the data set based on principle component analysis.*

| In-frame mutant data set | clpC t_0_ BR3, clpP t_0_ BR1, clpP t_10_ BR1, clpP t_30_ BR1, clpP t_0_ BR2, clpP t_10_ BR2, clpP t_30_ BR2 |
| --- | --- |
| Conditional mutant data set | wildtype t_0_ BR1, wild-type t_30_ BR3, *clpX* t_10_ BR3 and *con clpX* ctrl BR3 |

1. Gerth U, Kirstein J, Mostertz J, Waldminghaus T, Miethke M, Kock H, et al. Fine-tuning in regulation of Clp protein content in *Bacillus subtilis*. J Bacteriol. 2004 Jan;186(1):179–91.

2. Jumper J, Evans R, Pritzel A, Green T, Figurnov M, Ronneberger O, et al. Highly accurate protein structure prediction with AlphaFold. Nature. 2021 Aug;596(7873):583–9.

3. Pettersen EF, Goddard TD, Huang CC, Meng EC, Couch GS, Croll TI, et al. UCSF ChimeraX : Structure visualization for researchers, educators, and developers. Protein Sci. 2021 Jan;30(1):70–82.
